# Integrated Multivariate Predictive Model of Body Composition and Lipid Profile for Cardiovascular Risk Assessment

**DOI:** 10.3390/jcm14030781

**Published:** 2025-01-25

**Authors:** Belén Leyva-Vela, Maria Martínez-Olcina, Nuria Asencio-Mas, Manuel Vicente-Martínez, Bernardo José Cuestas-Calero, Piotr Matłosz, Alejandro Martínez-Rodríguez

**Affiliations:** 1 Department of Health, Vinalopó University Hospital, 03293 Elche, Spain; bmleyva@vinaloposalud.com; 2 Department of Analytical Chemistry, Nutrition and Food Science, University of Alicante, 03690 Alicante, Spain; maria.martinezolcina@ua.es (M.M.-O.); nuria.asencio@ua.es (N.A.-M.); mvmr2@alu.ua.es (M.V.-M.); amartinezrodriguez@ua.es (A.M.-R.); 3 Faculty of Health Sciences, Universidad Europea de Valencia, 46010 Valencia, Spain; bernardojose.cuestas@universidadeuropea.es; 4 Faculty of Physical Culture Sciences, Collegium Medicum, University of Rzeszów, 35-959 Rzeszów, Poland; 5 Alicante Institute for Health and Biomedical Research (ISABIAL), 03010 Alicante, Spain

**Keywords:** cardiovascular risk, lipid profile, body composition

## Abstract

**(1) Background/Objectives:** Cardiovascular diseases (CVD) are the leading cause of morbidity and mortality globally, necessitating effective risk prediction strategies. This study was aimed at developing and validating a multivariate predictive model integrating body composition and lipid profile to assess cardiovascular risk in an adult population. **(2) Methods:** A cross-sectional analysis of 90 participants from the general Spanish population was conducted. Participants were classified into cardiovascular risk groups (low, medium, high) based on systolic blood pressure. **(3) Results:** Descriptive and multinomial logistic regression analyses revealed significant associations between cardiovascular risk and specific parameters, such as visceral fat, glucose levels, and waist-to-hip ratio. Visceral adiposity emerged as a strong predictor of high cardiovascular risk, highlighting its critical role in cardiovascular health. Glucose levels were also significantly associated with increased risk, underscoring the importance of metabolic health in cardiovascular outcomes. Contrary to expectations, lipid markers like cholesterol and triglycerides did not show significant variations across risk categories, suggesting that traditional lipid profiles may not fully capture cardiovascular risk in the study group. Waist-to-hip ratio showed significant associations with cardiovascular risk transitions, particularly between low and medium risk, emphasizing the importance of fat distribution patterns. **(4) Conclusions:** These findings suggest that body composition, particularly visceral fat, is a crucial determinant of cardiovascular risk, necessitating more personalized risk assessment approaches that move beyond traditional lipid markers.

## 1. Introduction

Cardiovascular diseases (CVD) remain the leading cause of morbidity and mortality worldwide, contributing significantly to the global burden of non-communicable diseases (NCDs) [1,2,3]. According to the World Health Organization (WHO), CVDs are responsible for approximately 17.9 million deaths annually, accounting for nearly one-third of all global deaths [4,5]. This substantial burden underscores the need for effective prevention and management strategies to reduce the impact of CVDs on both individuals and healthcare systems [3]. Key strategies include the early identification of modifiable risk factors that can inform targeted interventions.

Among these risk factors, body composition and lipid profiles have consistently been shown to play critical roles in determining cardiovascular risk. Overweight and obesity, typically measured through body mass index (BMI), are major contributors to CVD, with excess adiposity linked to adverse metabolic changes that promote atherosclerosis [6,7,8]. In addition to overall adiposity, the distribution of body fat, particularly abdominal adiposity, as measured by visceral fat and waist-to-hip ratio, is recognized as a powerful independent predictor of cardiovascular risk [9]. Emerging tools, such as impedance at 50 kHz, offer additional insights into body composition by assessing parameters like total body water and lean mass, which may contribute to cardiovascular risk stratification in specific populations [10,11]. These findings highlight the importance of central fat accumulation and body composition analysis in the pathogenesis of CVD [7,12,13].

Lipid profiles, encompassing markers such as total cholesterol, low-density lipoprotein (LDL), high-density lipoprotein (HDL), and triglycerides, are another crucial determinant of cardiovascular health. Elevated levels of LDL cholesterol and triglycerides, alongside decreased HDL cholesterol, have been associated with an increased risk of atherosclerotic plaque formation, ultimately contributing to myocardial infarction and stroke [14,15,16]. However, recent evidence suggests that traditional lipid markers alone may not fully capture the nuances of cardiovascular risk, particularly in populations with varying degrees of adiposity.

While the individual impact of these risk factors has been well-documented, there remains a critical need for integrated tools that can jointly assess their contribution to cardiovascular risk. Multivariable predictive models provide an opportunity to evaluate how these factors interact and collectively influence the risk of developing CVD. Few studies, however, have focused on developing predictive models that simultaneously incorporate body composition and lipid profile to provide a comprehensive evaluation of cardiovascular risk.

This study aims to address this gap by developing and validating a multivariable predictive model that integrates body composition and lipid profile to assess cardiovascular risk in an adult population. By combining these critical determinants, the model provides healthcare professionals with a more nuanced and accurate tool for cardiovascular risk assessment, supporting personalized and effective prevention strategies to reduce the burden of CVD. Unlike many previous studies, this research uniquely focuses on the general population, rather than individuals with pre-existing conditions, offering broader insights into cardiovascular risk stratification. This approach ensures that the findings are applicable to diverse demographic groups and reinforce the importance of preventive strategies at the population level.

## 2. Materials and Methods

### 2.1. Study Design and Participants

This cross-sectional study included 90 participants, representing data from individuals recruited from the general adult population in Alicante, Spain. Participants were not part of any specific social or occupational group, nor were they selected based on any illness. Recruitment was conducted through advertisements and posters displayed in a variety of community hubs, including universities, health clinics, gyms, and shopping centers. These materials prominently highlighted the opportunity for participants to receive free health assessments, which encouraged participation. Interested individuals contacted the research team and underwent a preliminary screening to confirm their eligibility. Inclusion criteria required individuals to: (1) be 18 years or older; (2) have complete anthropometric and biochemical data available; (3) be free from any acute illness or condition that might interfere with the measurements; (4) provide informed consent for participation. Exclusion criteria include: (1) presence of chronic or acute illnesses that could significantly influence cardiovascular risk, such as diagnosed cardiovascular disease, diabetes, or cancer; (2) use of medications known to affect body composition or cardiovascular markers (e.g., diuretics, beta-blockers, or lipid-lowering drugs); (3) pregnancy or breastfeeding, given their potential influence on body composition and cardiovascular parameters; (4) incomplete or missing data in the required assessments.

Participants were then classified into three cardiovascular risk groups (low, medium, high) based on established clinical markers. This selection process aimed to ensure a representative sample of the general population while minimizing confounding factors that could influence the study outcomes.

### 2.2. Ethical Approval

This study was conducted in accordance with the Declaration of Helsinki and received ethical approval from the Ethics Committee of Alicante University (UA-2021-03-11). All participants provided informed consent prior to their inclusion in the study, ensuring their voluntary participation and understanding of the research procedures.

### 2.3. Body Composition and Anthopometric Assessment

Body composition was assessed using bioimpedance analysis with the BIODY XPERT^ZM^ (Aminogram, Marsella, France) and TANITA BC-730F (TANITA Corporation, Tokio, Japan) devices. Body mass index (BMI) was calculated as weight divided by height squared (kg/m^2^). Waist and hip circumferences were measured using a non-elastic measuring tape, and the waist-to-hip ratio was subsequently calculated.

### 2.4. Biochemical Analysis

Blood samples were drawn. Triglycerides, cholesterol and glucose levels were assessed using an Accutrend^®^ Plus (Roche Diagnostics, Mannheim, Germany). The results were measured in milligrams per deciliter (mg/dL). The Accutrend^®^ Plus test employs capillary serum and is based on the separation of blood cells through filtration with fiberglass when a blood drop is placed on the reactive strip. An enzymatic reaction within the strip occurs upon exposure to oxygen, resulting in a color change. The strip’s reflectance is measured at 660 nm, and the concentration of the various circulating parameters is determined using a straightforward algorithm. The accuracy of the Accutrend^®^ Plus, as stated in the product documentation, is 3.4%. Blood pressure (systolic and diastolic) was measured using a digital sphygmomanometer (Omron Healthcare, Osaka, Japan) after a 10-min rest in a seated position.

### 2.5. Statistical Analysis

Descriptive analyses were conducted to characterize the study population across cardiovascular risk categories (low, medium, high). The Shapiro–Wilk test was performed to establish the normality distribution. Continuous variables are presented as means and standard deviations, while categorical variables are described using frequencies and percentages. Comparisons across risk categories were performed using one-way analysis of variance (ANOVA) for normally distributed continuous variables. Where significant differences were identified, Tukey’s post-hoc tests were conducted to determine between-group differences. For non-normally distributed variables, Games–Howell tests were used.

To explore the relationships between variables, Pearson correlation coefficients were calculated, focusing on associations relevant to cardiovascular risk. Only statistically significant correlations (*p* < 0.05) are reported, highlighting the most impactful relationships. The outcome variable, cardiovascular risk, was categorized into three levels: low, medium, and high, based on systolic blood pressure (SBP) values.

SBP was selected as the differentiating criterion for cardiovascular risk groups due to its strong predictive value for cardiovascular events, as established in previous studies [17,18]. Elevated blood pressure is widely recognized as an independent risk factor for cardiovascular diseases and provides a robust and immediate marker for stratifying risk. Unlike other markers, SBP is a robust and widely accepted independent risk factor for cardiovascular diseases, including stroke, heart failure, and coronary artery disease. Its non-invasive measurement reflects arterial stiffness and cardiac workload, making it both practical and clinically relevant. Additionally, SBP offers consistent thresholds aligned with recommendations from organizations such as the American Heart Association (AHA), facilitating comparability with other studies and enhancing the translational value of the findings [18].

The categorization criteria were as follows: low risk (SBP < 120 mmHg), medium risk (120 mmHg ≤ SBP ≤ 139 mmHg), and high risk (SBP > 139 mmHg). These cutoff points were based on international guidelines, such as those from the American Heart Association (AHA), which identify these values as critical predictors of cardiovascular disease risk [17]. This categorical variable enabled the evaluation of cardiovascular risk as a multinomial outcome in subsequent analyses.

Multinomial logistic regression analysis was used to develop a predictive model for cardiovascular risk categories. Predictors included BMI, fat mass percentage, visceral fat, waist-to-hip ratio, cholesterol, triglycerides, and impedance at 50 kHz. Model performance was evaluated using pseudo R^2^ values (McFadden’s and Nagelkerke’s) to assess the explained variance, and odds ratios (ORs) with 95% confidence intervals (CIs) were used to interpret the contribution of each predictor.

An analysis of variance (ANOVA) was conducted to determine statistically significant differences in key variables, including BMI, fat mass percentage, visceral fat, waist-to-hip ratio, cholesterol, triglycerides, and impedance at 50 kHz across cardiovascular risk groups. Where significant differences were found, post-hoc analyses were conducted using Tukey’s adjustment to identify specific between-group differences. All statistical analyses were performed using Jamovi (Version 2.6.17.0, Sydney, Australia) with significance set at *p* < 0.05.

## 3. Results

Descriptive analyses were conducted to summarize the characteristics of the sample and explore differences across cardiovascular risk categories (low, medium, high). Table 1 presents the descriptive characteristics of the participants by cardiovascular risk level.

Body mass index (BMI) increased across cardiovascular risk groups, with mean values of 28.22 ± 2.18 kg/m^2^ in the low-risk group, 29.92 ± 4.05 kg/m^2^ in the medium-risk group, and 30.73 ± 3.54 kg/m^2^ in the high-risk group. Similarly, fat mass (%) exhibited a slight upward trend, with mean values ranging from 31.87 ± 9.05% in the low-risk group to 32.21 ± 11.12% in the high-risk group. Visceral fat presented more pronounced differences, with mean values of 7.88 ± 2.78 units in the low-risk group, 9.09 ± 4.14 units in the medium-risk group, and 11.36 ± 4.78 units in the high-risk group. The waist-to-hip ratio followed a similar pattern, increasing from 0.918 ± 0.187 in the low-risk group to 1.089 ± 0.135 in the high-risk group.

Skeletal muscle mass showed variability across groups, with the highest mean observed in the medium-risk group (30.93 ± 7.35 kg) compared to the low-risk group (27.81 ± 5.42 kg) and a slight decrease in the high-risk group (29.82 ± 6.53 kg).

Diastolic blood pressure (DBP) demonstrated significant increases across the risk groups. DBP increased from 70.59 ± 8.96 mmHg in the low-risk group to 85.79 ± 11.58 mmHg in the high-risk group. Pulse pressure (PPM) remained relatively consistent across the groups, with mean values between 70.61 ± 12.41 mmHg and 72.21 ± 8.03 mmHg.

Cholesterol levels were slightly higher in the medium-risk group (189.34 ± 21.45 mg/dL) compared to the low-risk group (185.59 ± 21.23 mg/dL) but decreased in the high-risk group (183.29 ± 10.34 mg/dL). Triglycerides followed a downward trend, with the lowest levels observed in the high-risk group (168.50 ± 19.21 mg/dL) compared to the low-risk group (187.44 ± 75.05 mg/dL).

Resistance (Ohm) at 50 kHz decreased progressively across the groups, with mean values of 544.56 ± 76.61 Ohm in the low-risk group, 516.10 ± 82.70 Ohm in the medium-risk group, and 509.25 ± 79.95 Ohm in the high-risk group. Similarly, reactance at 50 kHz followed the same trend, with values of 59.06 ± 12.19 Ohm in the low-risk group, 57.27 ± 9.04 Ohm in the medium-risk group, and 54.86 ± 10.22 Ohm in the high-risk group.

Correlation analyses were performed to explore the relationships between anthropometric, cardiovascular, biochemical, and bioelectrical impedance variables. The results of the Pearson correlation coefficients are presented in the correlation matrix (Table 2).

Body mass index (BMI) showed a significant positive correlation with fat mass (r = 0.579, *p* < 0.001), visceral fat (r = 0.691, *p* < 0.001), and waist-to-hip ratio (r = 0.333, *p* = 0.005). A weaker but significant correlation was observed between BMI and systolic blood pressure (SBP) (r = 0.294, *p* = 0.008) and diastolic blood pressure (DBT) (r = 0.381, *p* < 0.001). No significant correlations were found between BMI and triglycerides, glucose, or cholesterol levels.

Visceral fat was strongly associated with several variables, including BMI (r = 0.691, *p* < 0.001), DBP (r = 0.414, *p* < 0.001), waist-to-hip ratio (r = 0.351, *p* = 0.003), and skeletal muscle mass (r = 0.360, *p* < 0.001). Additionally, visceral fat exhibited a moderate correlation with fat mass (r = 0.237, *p* = 0.024) and SBP (r = 0.345, *p* = 0.002).

Waist-to-hip ratio was significantly correlated with DBP (r = 0.423, *p* < 0.001), SBP (r = 0.406, *p* < 0.001), and BMI (r = 0.333, *p* = 0.005). It also demonstrated moderate associations with fat mass (r = 0.351, *p* = 0.003) but did not correlate with glucose, triglycerides, or cholesterol.

SBP and DBP were strongly intercorrelated (r = 0.643, *p* < 0.001). SBP also showed moderate correlations with glucose levels (r = 0.359, *p* = 0.001) and weak positive associations with BMI and waist-to-hip ratio. Similarly, DBP was positively correlated with glucose (r = 0.254, *p* = 0.023).

Bioelectrical impedance variables revealed notable associations. Resistance at 50 kHz was negatively correlated with fat mass (r = −0.617, *p* < 0.001) and visceral fat (r = −0.297, *p* = 0.005), while reactance at 50 kHz was positively associated with skeletal muscle mass (r = 0.297, *p* = 0.005). Skeletal muscle mass also showed a strong inverse correlation with fat mass (r = −0.579, *p* < 0.001) and resistance at 50 kHz (r = −0.848, *p* < 0.001).

Triglycerides and cholesterol did not exhibit significant correlations with most variables, except for a weak positive association between glucose and cholesterol (r = 0.230, *p* = 0.040).

**Figure 1:**
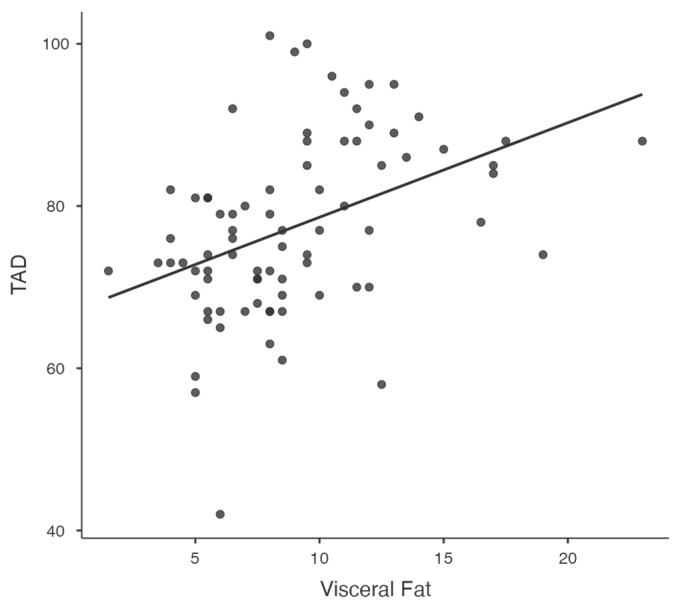
Scatterplot showing the correlation between visceral fat and diastolic blood pressure (DBP).

**Figure 2:**
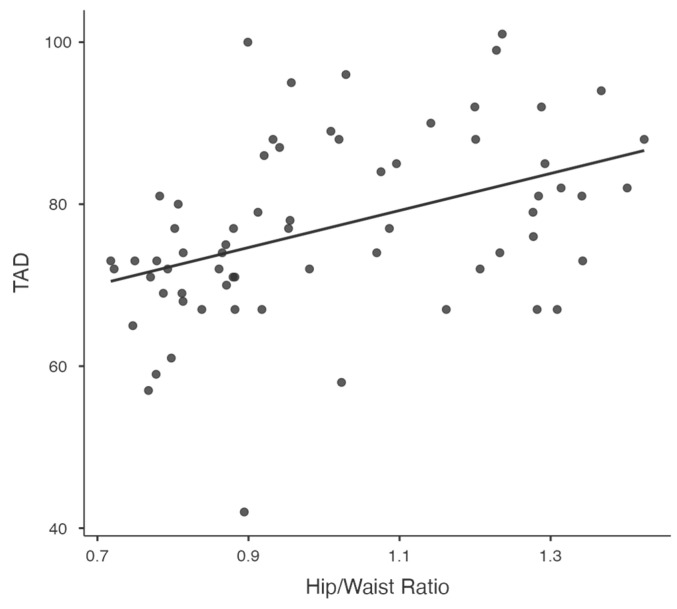
Scatterplot showing the correlation between waist-to-hip ratio and diastolic blood pressure (DBP).

**Figure 3:**
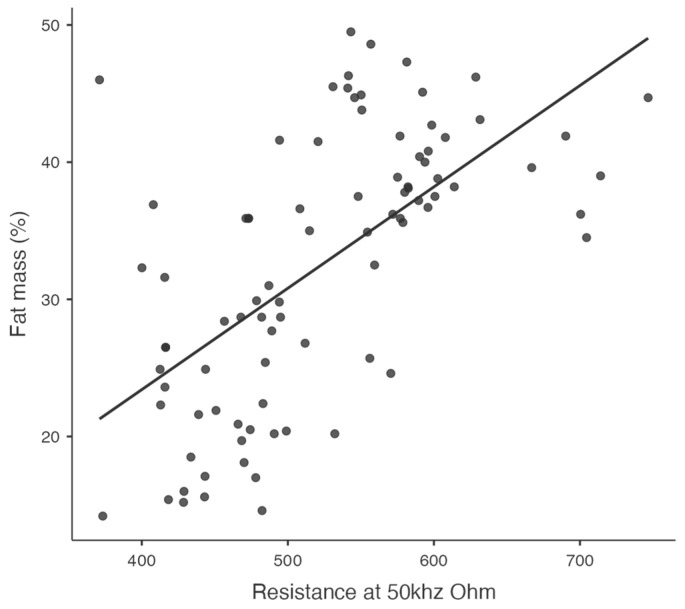
Scatterplot showing the correlation between resistance at 50 kHz and fat mass percentage.

**Figure 4:**
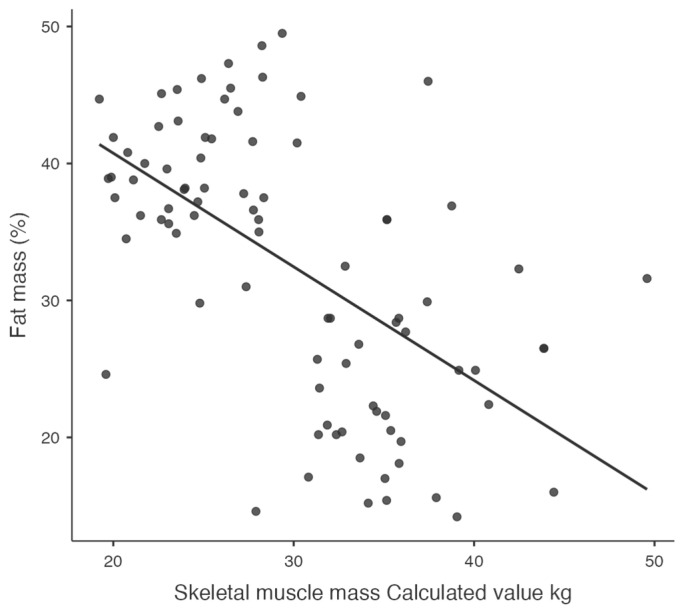
Scatterplot showing the inverse correlation between skeletal muscle mass and fat mass percentage.

Significant relationships between key variables, such as visceral fat and diastolic blood pressure (Figure 1), waist-to-hip ratio and diastolic blood pressure (Figure 2), and resistance at 50 kHz with fat mass percentage (Figure 3), are further illustrated in the scatterplots. Additionally, the inverse relationship between skeletal muscle mass and fat mass percentage is depicted in Figure 4. These visualizations complement the correlation matrix (Table 2) by providing an intuitive representation of the associations described.

The global multinomial logistic regression model (Table 3) revealed that diastolic blood pressure (DBP) and glucose levels were significant predictors of cardiovascular risk across both comparisons: medium vs low risk (OR = 1.13, *p* = 0.007 and OR = 1.17, *p* = 0.012, respectively) and high vs low risk (OR = 1.16, *p* = 0.008 and OR = 1.20, *p* = 0.013, respectively). Additionally, resistance at 50 kHz showed a significant inverse association with high vs low risk (OR = 0.95, *p* = 0.011), suggesting a potential protective effect.

Body mass index (BMI), fat mass, visceral fat, cholesterol, and triglycerides did not show significant associations with cardiovascular risk categories in the global model (all *p* > 0.05). However, glucose emerged as a consistent predictor across both comparisons.

The sex-specific analysis highlighted a significant difference, with females being substantially more likely to belong to the high-risk group compared to males (OR = 57.62, *p* < 0.001). This finding underscores potential biological or contextual differences in cardiovascular risk profiles between sexes.

No significant variations in the role of other predictors, such as BMI, BDP, or fat distribution, were observed when stratifying by sex, indicating that these variables exerted a consistent influence regardless of sex.

An analysis of variance (ANOVA) was conducted to assess differences in various predictors across cardiovascular risk groups (low, medium, high). These predictors included body mass index (BMI), diastolic bloor pressure (DBP), pulse pressure (PPM), fat mass (%), visceral fat, cholesterol, triglycerides, glucose, impedance at 50 kHz (Z50 Ohm), and waist-to-hip ratio. Significant differences were identified for some variables, indicating their varying impact on cardiovascular risk.

**Figure 5:**
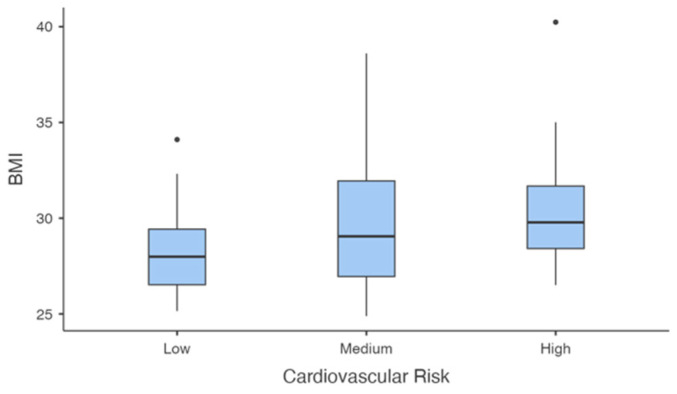
Distribution of Body Mass Index (BMI = kg/m^2^) across Cardiovascular Risk levels.

Significant differences in BMI (Figure 5) were observed across cardiovascular risk groups (F(2, 31.4) = 4.396, *p* = 0.021). Post-hoc tests revealed that the high-risk group had significantly higher BMI compared to the low-risk group (mean difference = −2.50, *p* = 0.048), while no significant differences were observed between the medium- and low-risk groups (*p* = 0.096) or between the medium- and high-risk groups (*p* = 0.726).

**Figure 6:**
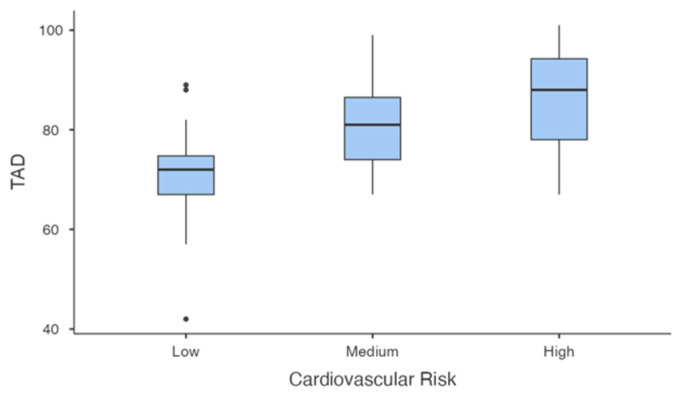
Diastolic blood pressure (DBP) by Cardiovascular Risk group.

DBP (Figure 6) varied significantly across groups (F(2, 33.1) = 16.152, *p* < 0.001). Post-hoc comparisons using the Games–Howell test revealed that DBP was significantly higher in the high-risk group compared to the low-risk group (mean difference = 15.20, *p* < 0.001) and in the medium-risk group compared to the low-risk group (mean difference = 10.4, *p* < 0.001). However, no significant differences were observed between the medium- and high-risk groups (mean difference = 4.75, *p* = 0.367).

**Figure 7:**
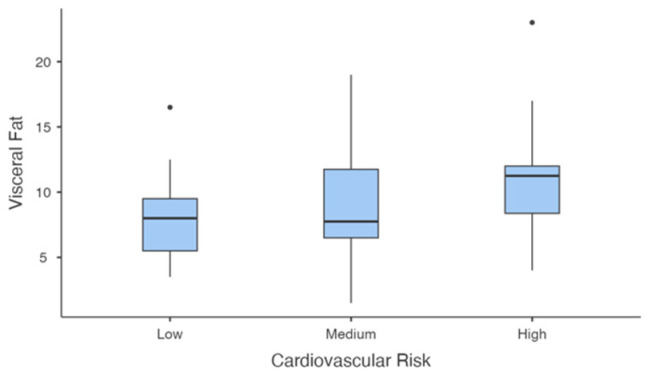
Visceral Fat levels Across Cardiovascular Risk groups.

Visceral fat (Figure 7) showed significant differences across risk groups (F(2, 31.4) = 3.593, *p* = 0.039). The high-risk group had significantly higher visceral fat compared to the low-risk group (mean difference = −3.47, *p* = 0.013). However, differences between the medium- and low-risk groups and between the medium- and high-risk groups were not significant (*p* = 0.393 and *p* = 0.150, respectively).

**Figure 8:**
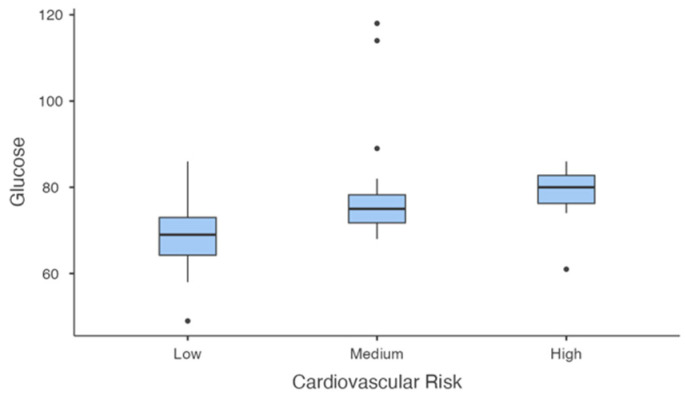
Glucose levels Across Cardiovascular Risk groups.

Glucose levels (Figure 8) were significantly different across groups (F(2, 40.5) = 13.233, *p* < 0.001). Post-hoc tests revealed that glucose levels were significantly higher in the high-risk group compared to the low-risk group (mean difference = −9.92, *p* = 0.002) and in the medium-risk group compared to the low-risk group (mean difference = −9.05, *p* < 0.001). No significant differences were observed between the medium- and high-risk groups (*p* = 0.951).

**Figure 9:**
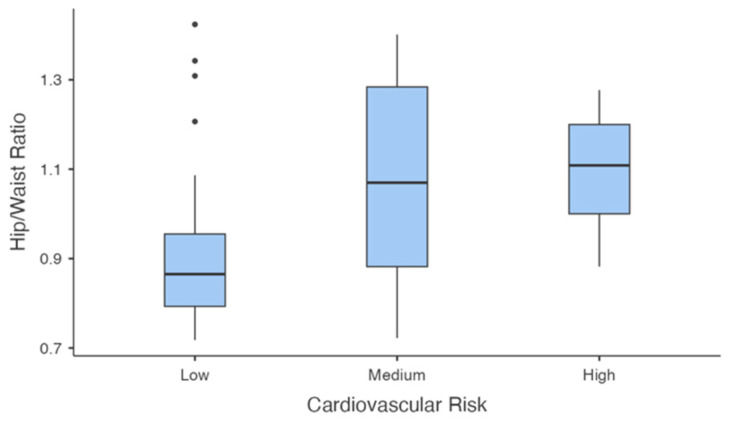
Waist-to-Hip Ratio Across Cardiovascular Risk Groups.

Waist-to-hip ratio (Figure 9) also differed significantly across groups (F(2,35.1) = 6.492, *p* = 0.004). Post-hoc comparisons using the Games–Howell test revealed that the medium-risk group had a significantly higher ratio compared to the low-risk group (mean difference = −0.157, *p* = 0.021), as did the high-risk group compared to the low-risk group (mean difference = −0.171, *p* = 0.007). No significant difference was observed between the medium- and high-risk groups (mean difference = −0.014, *p* = 0.970).

No significant differences were found for fat mass (%), cholesterol, triglycerides, impedance at 50 kHz, reactance at 50 kHz, or skeletal muscle mass (all *p* > 0.05).

## 4. Discussion

This study aimed to investigate the relationship between body composition, lipid profiles, and cardiovascular risk in a general population sample. The findings highlight that specific body composition markers, including diastolic blood pressure (DBP), visceral fat, glucose levels, and waist-to-hip ratio, are significantly associated with cardiovascular risk, while traditional lipid markers, such as cholesterol and triglycerides, did not vary significantly across risk groups. These results emphasize the importance of incorporating emerging and non-traditional markers into cardiovascular risk stratification.

Visceral fat emerged as a strong predictor of cardiovascular risk, particularly for individuals in the high-risk category. Unlike subcutaneous fat, visceral adiposity is metabolically active, contributing to insulin resistance, systemic inflammation, and adverse lipid profiles, all of which promote cardiovascular disease development. These findings reinforce existing evidence linking visceral fat to heightened cardiovascular risk and stress the need for targeted interventions to reduce visceral adiposity as a preventive strategy [4,8,19,20].

Glucose levels were significantly higher in the medium- and high-risk groups compared to the low-risk group. Elevated glucose is a hallmark of metabolic dysregulation and insulin resistance, which are closely tied to cardiovascular health. These results underscore the importance of managing glucose levels as part of an integrated strategy for cardiovascular risk reduction [21,22].

The waist-to-hip ratio showed significant differences between low- and medium-risk groups and between low- and high-risk groups, further indicating its utility in identifying transitions in cardiovascular risk. This measure reflects both central and peripheral fat distribution, and its significant association with risk suggests that fat distribution patterns contribute to the development of cardiovascular disease. However, the lack of significant differences between medium- and high-risk groups may indicate that its predictive value diminishes at higher levels of risk [4,23,24].

Building on these findings, body composition metrics have consistently demonstrated significant associations with cardiovascular risk, offering valuable insights beyond traditional markers such as BMI. For instance, visceral adipose tissue, a key determinant of cardiovascular health, contributes to increased vascular stiffness and endothelial dysfunction due to its pro-inflammatory and pro-thrombotic properties [25]. Studies among middle-aged Slovak women with cardiovascular complications highlighted how higher waist-to-hip ratios and fat mass percentages were closely tied to cardiovascular disease, emphasizing the critical role of fat distribution [26]. Additionally, research on multi-ethnic populations revealed distinct patterns of adiposity, with variations in the impact of body fat and lean mass on cardiovascular risk markers such as systolic blood pressure and HbA1c, underscoring the importance of context-specific assessments [27]. Finally, recent evidence has also suggested the interplay between body composition and cardiometabolic risk factors, showing that factors such as adiposity and lean mass strongly influence blood pressure and lipid profiles [28]. These findings collectively highlight the importance of integrating body composition parameters into cardiovascular risk assessment for a more nuanced understanding of disease etiology and progression.

In contrast, body mass index (BMI), while traditionally used as a measure of overall adiposity, did not exhibit significant associations with cardiovascular risk across all groups. BMI’s inability to account for fat distribution may limit its predictive utility in this context. These results align with growing evidence suggesting that BMI should be complemented with more precise measures, such as visceral fat or waist-to-hip ratio, to better understand cardiovascular risk [7,21].

Traditional lipid markers, including cholesterol and triglycerides, did not differ significantly across cardiovascular risk groups. This lack of variation cannot be attributed to pharmacological interventions, as the study excluded individuals using medications known to affect lipid profiles, such as statins. It is possible that the relatively healthy profile of the participants, combined with unmeasured factors such as dietary habits or genetic predispositions, mitigated the expected associations between lipid markers and cardiovascular risk. Additionally, the reliance on total cholesterol or triglycerides may overlook more nuanced lipid abnormalities, such as LDL particle size or HDL function, which may better capture the complexities of cardiovascular risk [14,16,29,30,31,32]. These findings highlight the need for future research to explore the influence of these variables in greater detail.

The findings for impedance at 50 kHz (Z50 Ohm) were less definitive, as no significant differences were observed across risk groups. This result suggests that while impedance-based measures provide valuable information about body composition, they may be less sensitive markers for differentiating cardiovascular risk levels in this population [10,11,29,32].

This study’s strengths lie in its comprehensive assessment of both traditional and emerging markers of cardiovascular risk, including direct measures of body composition. Examining multiple facets of adiposity and glucose levels provides a nuanced perspective on cardiovascular risk. However, the cross-sectional design limits causal inferences, and the reliance on bioimpedance, while practical, may be less accurate than imaging techniques such as MRI or DEXA. Nonetheless, this study ensured controlled and standardized conditions during all measurements, including adequate participant hydration, to minimize potential sources of error and maximize data reliability. Future studies could benefit from incorporating advanced imaging modalities to validate and complement the findings.

Another limitation of this study is the absence of an assessment of chest wall conformation as part of the anthropometric evaluation of cardiovascular risk. Chest wall morphology, such as a concave-shaped chest wall measured by the Modified Haller Index (MHI), has been shown to influence cardiovascular outcomes, with recent evidence indicating a significantly lower probability of adverse cardiovascular events in individuals with concave chest walls compared to those with normal chest shapes over a medium-term follow-up period [31]. This highlights the potential value of incorporating simple, non-invasive assessments of chest wall conformation, such as echocardiographic measurements, into cardiovascular risk stratification. Future studies should include additional measures, such as thoracic circumference or sternal angle assessments, to provide a more comprehensive understanding of the relationship between body structure and cardiovascular health.

These findings highlight the importance of incorporating measures of visceral fat, glucose, and body composition into routine cardiovascular risk assessments. Traditional lipid markers alone may not adequately capture risk profiles, particularly in populations with diverse adiposity distributions. By focusing on a general population, this study provides valuable insights that can guide public health interventions and risk assessment strategies on a broader scale. Future research should aim to validate these findings through longitudinal analyses, explore advanced lipid profiling, and investigate novel methods such as the integration of chest wall morphology into cardiovascular risk models. Leveraging open data sharing and collaborative research will further enhance reproducibility and innovation in cardiovascular risk management.

## 5. Conclusions

This study highlights the significant association of visceral fat with cardiovascular risk, particularly for individuals in the high-risk category. These findings reinforce the importance of incorporating measures of visceral adiposity into cardiovascular risk assessments. In contrast, traditional lipid markers, such as cholesterol and triglycerides, showed no significant associations, suggesting the need for alternative approaches to stratify cardiovascular risk.

Further research is warranted to validate these findings in larger populations and explore the potential for integrating body composition metrics into routine clinical practice for improved cardiovascular risk management.

## Figures and Tables

**Table 1:** Descriptive Characteristics of the Sample by Cardiovascular Risk Level.

Variable	Low Risk (*n* = 34)	Medium Risk (*n* = 32)	High Risk (*n* = 14)
BMI (kg/m^2^)	28.22 ± 2.18	29.92 ± 4.05	30.73 ± 3.54
Fat Mass (%)	31.87 ± 9.05	31.41 ± 9.78	32.21 ± 11.12
Visceral Fat (units)	7.88 ± 2.78	9.09 ± 4.14	11.36 ± 4.78
Waist-to-Hip Ratio	0.918 ± 0.187	1.075 ± 0.223	1.089 ± 0.135
Cholesterol (mg/dL)	185.59 ± 21.23	189.34 ± 21.45	183.29 ± 10.34
Triglycerides (mg/dL)	187.44 ± 75.05	180.63 ± 52.73	168.50 ± 19.21
Skeletal Muscle Mass (kg)	27.81 ± 5.42	30.93 ± 7.35	29.82 ± 6.53
SBP (mmHg)	109.82 ± 6.60	127.22 ± 5.63	149.36 ± 5.56
DBP (mmHg)	70.59 ± 8.96	81.03 ± 8.33	85.79 ± 11.58
Pulse Pressure (mmHg)	71.24 ± 10.83	70.61 ± 12.41	72.21 ± 8.03
Resistance (Ohm, 50 kHz)	544.56 ± 76.61	516.10 ± 82.70	509.25 ± 79.95
Reactance (Ohm, 50 kHz)	59.06 ± 12.19	57.27 ± 9.04	54.86 ± 10.22

Kg = kilograms; m = meters; % = percentage; mg = milligrams; dL = deciliters; mmHg = millimeters of mercury; kHz = kilohertz; SBP = systolic blood pressure; DBP = diastolic blood pressure. Note: Values are presented as mean ± standard deviation.

**Table 2:** Correlation Matrix of Key Variables.

	BMI	SBP	DBP	PPM	FM%	VF	WHR	CHOL	TG	GLU	RES_50	REA_50	SMM
**BMI**	—	0.294 **	0.381 ***	0.131	0.579 ***	0.691 ***	0.333 **	−0.047	0.021	0.078	−0.189	−0.124	0.184
**SBP**	0.294 **	—	0.643 ***	−0.025	0.040	0.345 **	0.406 ***	−0.046	−0.096	0.359 **	−0.198	−0.226 *	0.136
**DBP**	0.381 ***	0.643 ***	—	0.125	0.124	0.414 ***	0.423 ***	0.126	−0.014	0.254 *	−0.106	−0.217	0.137
**PPM**	0.131	−0.025	0.125	—	0.277 *	0.036	0.071	0.015	−0.015	0.085	0.288 *	0.094	−0.279 *
**FM%**	0.579 ***	0.040	0.124	0.277 *	—	0.237 *	0.351 **	0.053	0.081	−0.041	0.617 ***	0.366 ***	−0.579 ***
**VF**	0.691 ***	0.345 **	0.414 ***	0.036	0.237 *	—	0.113	0.094	0.014	0.102	−0.297 **	−0.264 *	0.360 ***
**WHR**	0.333 **	0.406 ***	0.423 ***	0.071	0.351 **	0.113	—	0.029	−0.019	0.111	0.213	0.173	−0.069
**CHOL**	−0.047	−0.046	0.126	0.015	0.053	0.094	0.029	—	0.145	0.230 *	0.120	0.002	0.032
**TG**	0.021	−0.096	−0.014	−0.015	0.081	0.014	−0.019	0.145	—	−0.193	0.008	−0.021	0.001
**GLU**	0.078	0.359 **	0.254 *	0.085	−0.041	0.102	0.111	0.230 *	−0.193	—	−0.033	−0.068	0.074
**RES_50**	−0.189	−0.198	−0.106	0.288 *	0.617 ***	−0.297 **	0.213	0.120	0.008	−0.033	—	0.560 ***	−0.848 ***
**REA_50**	−0.124	−0.226 *	−0.217	0.094	0.366 ***	−0.264 *	0.173	0.002	−0.021	−0.068	0.560 ***	—	0.297 **
**SMM**	0.184	0.136	0.137	−0.279 *	−0.579 ***	0.360 ***	−0.069	0.032	0.001	0.074	−0.848 ***	0.297 **	—

BMI = Body Mass Index; SBP = Systolic Blood Pressure; DBT = Diastolic Blood Pressure; PPM = Pulse Pressure; FM% = Fat Mass Percentage; VF = Visceral Fat; WHR = Waist-to-Hip Ratio; CHOL = Cholesterol; TG = Triglycerides; GLU = Glucose; RES_50 = Resistance at 50 kHz; REA_50 = Reactance at 50 kHz; SMM = Skeletal Muscle Mass. * *p* < 0.05, ** *p* < 0.01, *** *p* < 0.001. Values represent Pearson’s r correlations.

**Table 3:** Multinomial Logistic Regression Analysis for Predicting Cardiovascular Risk Categories.

Predictor	Medium vs. Low Risk (OR [95% CI])	*p*-Value	High vs. Low Risk (OR [95% CI])	*p*-Value	Interpretation
BMI	1.18 [0.72, 1.94]	0.499	1.14 [0.61, 2.13]	0.692	Not significant
DBP	1.13 [1.03, 1.23]	0.007	1.16 [1.04, 1.30]	0.008	Significant predictor for both risks
PPM	0.99 [0.92, 1.06]	0.760	1.00 [0.91, 1.09]	0.966	Not significant
FM%	0.97 [0.79, 1.20]	0.777	0.91 [0.68, 1.22]	0.536	Not significant
VF	0.91 [0.68, 1.21]	0.521	1.20 [0.84, 1.73]	0.311	Not significant
CHOL	0.99 [0.96, 1.04]	0.926	0.97 [0.91, 1.04]	0.392	Not significant
TG	1.00 [0.99, 1.01]	0.681	1.00 [0.98, 1.02]	0.993	Not significant
GLU	1.17 [1.04, 1.32]	0.012	1.20 [1.04, 1.38]	0.013	Significant predictor for both risks
RES_50	1.00 [0.97, 1.03]	0.931	0.95 [0.92, 0.99]	0.011	Protective for high risk
REA_50	0.97 [0.72, 1.31]	0.850	1.46 [0.94, 2.27]	0.096	Not significant
SMM	0.97 [0.75, 1.25]	0.797	0.81 [0.59, 1.11]	0.184	Not significant

BMI = Body Mass Index; DBP = Diastolic Blood Pressure; PPM = Pulse Pressure; FM% = Fat Mass Percentage; VF = Visceral Fat; CHOL = Cholesterol; TG = Triglycerides; GLU = Glucose; RES_50 = Resistance at 50 kHz; REA_50 = Reactance at 50 kHz; SMM = Skeletal Muscle Mass. OR = Odds Ratio, CI = Confidence Interval. *p* < 0.05 was considered statistically significant.

## Data Availability

Data presented in this study are available on request from the corresponding author. The data are not publicly available due to is personal health information.

## Abbreviations

The following abbreviations are used in this manuscript:

BMI	Body Mass Index
SBP	Systolic Blood Pressure
DBP	Diastolic Blood Pressure
PPM	Pulse Pressure
FM%	Fat Mass Percentage
VF	Visceral Fat
WHR	Waist-to-Hip Ratio
CHOL	Cholesterol
TG	Triglycerides
GLU	Glucose
RES_50	Resistance at 50 kHz
REA_50	Reactance at 50 kHz
SMM	Skeletal Muscle Mass
CVD	Cardiovascular Diseases
NCD	Non-Communicable Diseases
LDL	Low-Density Lipoprotein
HDL	High-Density Lipoprotein
AHA	American Heart Association
Z50	Impedance at 50 kHz
